# Dose-Dependent Effects of Morphine Exposure on mRNA and microRNA (miR) Expression in Hippocampus of Stressed Neonatal Mice

**DOI:** 10.1371/journal.pone.0123047

**Published:** 2015-04-06

**Authors:** Ryan M. McAdams, Ronald J. McPherson, Richard P. Beyer, Theo K. Bammler, Frederico M. Farin, Sandra E. Juul

**Affiliations:** 1 Department of Pediatrics, Division of Neonatology, University of Washington, Seattle, Washington, United States of America; 2 Dept of Environmental & Occupational Health Sciences, University of Washington, Seattle, Washington, United States of America; CNRS UMR7275, FRANCE

## Abstract

Morphine is used to sedate critically ill infants to treat painful or stressful conditions associated with intensive care. Whether neonatal morphine exposure affects microRNA (miR) expression and thereby alters mRNA regulation is unknown. We tested the hypothesis that repeated morphine treatment in stress-exposed neonatal mice alters hippocampal mRNA and miR expression. C57BL/6 male mice were treated from postnatal day (P) 5 to P9 with morphine sulfate at 2 or 5 mg/kg *ip* twice daily and then exposed to stress consisting of hypoxia (100% N_2_ 1 min and 100% O_2_ 5 min) followed by 2h maternal separation. Control mice were untreated and dam-reared. mRNA and miR expression profiling was performed on hippocampal tissues at P9. Overall, 2 and 5 mg/kg morphine treatment altered expression of a total of 150 transcripts (>1.5 fold change, P<0.05) from which 100 unique mRNAs were recognized (21 genes were up- and 79 genes were down-regulated), and 5 mg/kg morphine affected 63 mRNAs exclusively. The most upregulated mRNAs were fidgetin, arginine vasopressin, and resistin-like alpha, and the most down-regulated were defensin beta 11, aquaporin 1, calmodulin-like 4, chloride intracellular channel 6, and claudin 2. Gene Set Enrichment Analysis revealed that morphine treatment affected pathways related to cell cycle, membrane function, signaling, metabolism, cell death, transcriptional regulation, and immune response. Morphine decreased expression of *miR-204-5p*, *miR-455-3p*, *miR-448-5p*, and *miR-574-3p*. Nine morphine-responsive mRNAs that are involved in neurodevelopment, neurotransmission, and inflammation are predicted targets of the aforementioned differentially expressed miRs. These data establish that morphine produces dose-dependent changes in both hippocampal mRNA and miR expression in stressed neonatal mice. If permanent, morphine–mediated neuroepigenetic effects may affect long-term hippocampal function, and this provides a mechanism for the neonatal morphine-related impairment of adult learning.

## Introduction

Critically ill preterm and full-term infants in the neonatal intensive care unit (NICU) undergo inescapable stress, often for prolonged periods. These infants are commonly exposed to high doses of morphine to provide analgesia, sedation, and to treat neonatal abstinence syndrome [1]. The individual and combined effects of stress and morphine may affect neurodevelopment [2]. To evaluate these potential influences on developmental outcomes, we developed mouse and rat models of neonatal stress and morphine treatment to assess short- and long-term effects on development. The hippocampus is a logical focus because it mediates learning, it contains opioid receptors [3], and hippocampal mu-opioid receptor densities are altered by prenatal morphine [4] or adult immobilization stress [5].

A series of experiments identified that early stress and morphine produce lasting behavioral and hormonal effects including impairment of learning and increased sensitivity to hypoxia [6–10]. In addition, when we exposed neonatal mice to stress with and without morphine, we identified patterns of hippocampal mRNA expression associated with either morphine, stress, or the combination of morphine+stress. For example, neonatal stress and morphine elevated expression of mitochondrial electron transport-related gene sets, but down-regulated gene sets related to brain development and growth [11]. Morphine administration before and during pregnancy can cause cerebellar cortex thinning (Purkinje and internal granular layers) with Purkinje cell loss and decreased size of Purkinje cells in neonatal mice [12]. The mechanisms involved in regulating these morphine and stress-mediated changes in the developing brain remain unclear.

Morphine-mediated changes in hippocampal gene expression may involve regulation by miRs. miRs are single-stranded nonprotein-coding RNA transcripts that regulate gene expression by binding to the 3′ untranslated regions of mRNA transcripts to either inhibit mRNA translation or reduce mRNA stability [13]. More than 60% of mammalian mRNA transcripts are regulated by miRs [14]. Thousands of gene networks, many related to nervous system development and function, are regulated by miR activity [15–21]. A variety of morphine-modulated miRs are recognized with effects on addiction, brain development and immune function [22–25]. For example, *let-7* miRs regulate opioid tolerance by repressing translation of mu opioid receptor mRNA, *miR-154* and *miR-675* are increased in mice trained to self administer morphine [26, 27], morphine decreases *miR-133b* in immature rat hippocampal neuron cultures [28], and morphine increases *miR-15b* and decreases *miR-181b* in human monocyte-derived macrophages [29]. Since miRs regulate developmental gene expression and cellular functions, changes in miR expression secondary to morphine exposure may adversely impact neurodevelopment.

During critical care, a substantial number of premature infants are treated with morphine to reduce pain and stress and this raises concerns about the potential impact of stress and morphine on brain development [2]. We hypothesized that morphine may mediate changes in neonatal brain gene expression by altering miR-mediated mRNA regulation in stressed mice. If true, identifying whether neonatal morphine exposure affects miR-regulated pathways that may be deleterious to brain development is important. We describe the acute effects of neonatal morphine treatment on mRNA and miR expression in mouse hippocampus, and evaluate the miR-targeted mRNA pathways that are affected by neonatal morphine.

## Materials and Methods

### Animals

Adult wild-type C57BL/6 mice were purchased (Harlan, San Diego, CA) and housed under a 12 h light-dark cycle with free access to food and water. Breeding was performed with two females per cage. Birth was recorded as postnatal day (P) 1. Litters were culled to n = 7 maximum per dam. Mortality and weights were monitored. All animal procedures were approved by the University of Washington’s Animal Care and Use Committee.

### Treatments

Beginning on P5, male mice were assigned to one of 3 treatment conditions: untreated control (Con), morphine 2 mg/kg + stress (MS2), and morphine 5 mg/kg + stress (MS5). Untreated control animals were housed normally and exposed to minimal handling. Treated pups were exposed to stress, apnea, and morphine. To produce stress, mice were separated from the dam and isolated in cups within a veterinary warmer at 32°C (08:00 h to16:00 h). To simulate apnea, mice were exposed to 100% N_2_ for 1 min then 100% O_2_ for 5 min, twice daily (08:00 and 15:30 h). Just prior to each apnea exposure, mice received 10 μL *s*.*c*. injections of morphine. After treatments, mice were then returned to the home cage with the untreated control littermates and could nurse overnight *ad lib*. Experiments were repeated to acquire six animals per treatment condition.

### Tissue collection

All animals were killed on P9 by overdose with Beuthanasia-D (2.2 mL/kg, *i*.*p*.). After euthanasia, animals underwent cervical dislocation, brains were quickly removed, and bilateral hippocampi were dissected and pooled into a prelabeled tube that was immediately frozen in liquid nitrogen and then stored at -80°C until assay. RNA from the hippocampus was extracted using the Qiagen miRNeasy Kit protocol (Qiagen, Valencia, CA). RNA concentration was determined by measuring OD_260_ with a NanoDrop ND-1000 Spectrophotometer (Thermo Fisher Scientific, Waltham, MA). RNA purity was assessed by measuring OD_260/280_ and OD_260/230_ ratios. RNA integrity was evaluated using an Agilent 2100 Bioanalyzer (Agilent Technologies, Inc., Palo Alto, CA). Only RNA samples with 28S and 18S ribosomal RNA peaks that were baseline separated, and with OD_260/280_ and OD_260/230_ ratios of 1.8–2.1 were used for further analysis.

### Microarray processing and data analysis

RNA samples were prepared for hybridization onto Agilent mouse 8x60k mRNA microarrays (Agilent Technologies, Inc. Santa Clara, CA) using 200 ng of total RNA per sample and the manufacturer’s established protocols. RNA from 5 animals of each experimental group was arrayed on 15 arrays. Hybridization and washing of these arrays was accomplished using HS 400 Pro hybridization and wash stations (Tecan Systems, Inc., San Jose, CA) and scanned using an Agilent DNA Microarray Scanner (Agilent Technologies, Inc. Santa Clara, CA) using previously established methods. Raw microarray data were preprocessed with the Agilent Feature Extraction image analysis software (Agilent) and processed using Bioconductor *Agi4x44PreProcess* package with the *normexp* option for background adjustment and *quantile normalization* for inter-array correction [30]. Using the normalized data, we detected differential expression using Bioconductor’s limma software package, which calculates a p-value for each gene using a modified t-test in conjunction with an empirical Bayes method to moderate the standard errors of the estimated log-fold changes, and we estimated the false discovery rate using Bioconductor *p*.*adjust* [31–34].

Processing of the RNA samples for the Affymetrix GeneChip miR Array, which contains probe sets designated for various species including mouse, was performed according to the standard protocol recommended by the manufacturer (www.affymetrix.com/) using 500 ng of total RNA per sample. RNA from 3 animals of each experimental group were used for miR expression profiling on 9 arrays. Hybridized Affymetrix arrays were scanned with an Affymetrix GeneChip 3000 fluorescent scanner. Image generation and feature extraction was performed using Affymetrix GeneChip Command Console Software. Raw GeneChip miR array data was preprocessed with Affymetrix miR QCTool software (http://www.affymetrix.com/products_services/arrays/specific/mi_rna.affx#1_4). The preprocessing steps include the following: probe specific signal detection calls based on a Wilcoxon Rank-Sum test of the miR probe set signals compared to the distribution of signals from GC content matched antigenomic probes, background estimation and correction, constant variance stabilization on probes, probe level quantile normalization, and finally probe summarization using median polish. miR RNA arrays included oligo spike-in controls for monitoring array quality. Arrays were required to pass manufacturer’s quality control recommendations before further analysis. Only probes for *Mus musculus* were used in the subsequent analysis. miRs with significant evidence for differential expression were identified using the limma software. *P*-values were calculated with a modified *t* test with in conjunction with an empirical Bayes method to moderate the standard errors of the estimated Log-fold changes. *P*-values were adjusted for multiplicity with the Bioconductor package q value [35], which allows for selecting statistically significant miRs at a chosen estimated false discovery rate. P-values shown in text, tables and figures are unadjusted p-values unless otherwise stated. All samples and all expression values were used in the analysis of the data, that is, potential outliers were not removed from the analysis.

### Quantitative TaqMan based RT-PCR analysis of protein coding mRNA

Quantitative PCR (qPCR) analysis was performed to validate gene expression changes for the seven specific genes (*Aqp1*, *Avp*, *Calml4*, *Cldn2*, *Defb11*, *Fign*, *Renla*) that exhibited the greatest differential expression observed in microarray analysis. Reverse transcription was performed according to the manufacturer’s established protocol using total RNA and the SuperScript III First-Strand Synthesis System (Invitrogen, Carlsbad, CA, USA). For gene expression measurements, 2 μL of cDNA were included in a PCR reaction (12 μL final volume) that also consisted of the ABI inventoried TaqMan Gene Expression Assays mix and the TaqMan Gene Expression Master Mix according to the manufacturer’s protocol (Applied Biosystems, Inc., Foster City, CA, USA). Amplification and detection of PCR amplicons for *Avp* and *Cldn2* were performed with the ABI PRISM 7900 system (Applied Biosystems, Inc., Foster City, CA, USA), whereas the ROCHE LightCycler 480 was used for *Aqp1*, *Calml4*, *Defb1*, *Fign and Renla*. The following PCR reaction profile was used: 1 cycle of 95°C for 10 min, 40 cycles of 95°C for 15 s, and 60°C for 1 min. Glyceraldehyde-3-phosphate dehydrogenase (GAPDH) amplification plots derived from serial dilutions of an established reference sample were used to create a linear regression formula in order to calculate expression levels. GAPDH gene expression levels were utilized as an internal control to normalize the data. This was carried out independently for the data that was generated with the ABI PRISM 7900 system as well as the ROCHE LightCycler 480. The same 15 samples, each derived from a different mouse, were used for RT-PCR analysis and mRNA expression profiling. The normalized qPCR data were compared directly using t-tests and also via conventional linear model using morphine dose as a continuous independent variable. The following TaqMan RT-PCR assays from Life Technologies (Carlsbad, CA, USA) were used to assess expression of *Retnla*, *Defb11*, *Aqp1*, *Calml4*, *Fign*, *Cldn2*, *Avp* and *GAPDH*: *Retnla* Assay ID Mm00445109_m1, *Defb11* Assay ID Mm02029641_u1, *Aqp1* Assay ID Mm00431834_m1, *Calml4* Assay ID Mm01174378_m1, *Fign* Assay ID Mm01275471_m1, *Cldn2* Assay ID Mm00516703_s1, *Avp* Assay ID Mm00437761_g1, *GAPDH* Assay ID Mm99999915_g1.

### Quantitative TaqMan based RT-PCR analysis of miRs

Reverse transcription was performed using the Life Technologies TaqMan MicroRNA RT Kit following the manufacturer’s established protocol for multiplex RT for TaqMan MicroRNA Assays. In addition to the manufacturer’s standard reagents, 20ng of total RNA was used in conjunction with a multiplex RT primer pool (created by pooling TaqMan primers, drying down in a speed vacuum, and adjusting volume for a 62.5nM concentration for each primer). After Reverse Transcription was completed, Real-Time PCR was carried out on an ABI Prism 7900 Sequence Detection System (Life Technologies) with *U6* snRNA used as an endogenous control to normalize the miR RT-PCR data. RT-PCR analysis was performed for *miR-448*, *miR-34c*, miR-34c*, *miR-204*, *miR-1839-3p*, *miR-153*, *miR-1983*, *miR-214*, *miR-455*, *miR-574-3p*. In addition to the 9 samples that were used for miR array expression profiling, 4 additional biological replicates each for the 2 mg/kg and 5 mg/kg experimental groups, and 2 additional replicates for the 0 mg/kg control group were used for miR RT-PCR analysis, for a total of 19 samples. The following TaqMan RT-PCR assays from Life Technologies were used to assess expression of *mmu-miR-455*, *mmu-miR-574-3p*, *mmu-miR-448-5p*, *mmu-miR-45c-5p*, *mmu-miR-34c-3p*, *mmu-miR-204*, *mmu-miR-1839-3p*, *mmu-miR-153*, *mmu-miR-1983*, *mmu-miR-214* in mouse: 002455 (*mmu-miR-455*), 002349 (*mmu-miR-574-3p*), 464921_mat (*mmu-miR-448-5p*), 000428 (*mmu-miR-45c-5p*), 001197 (*mmu-miR-204*), 002584 (mmu-miR-34c*), 121203_mat (*mmu-miR-1839-3p*), 001191 (*mmu-miR-153*), 121204_mat (*mmu-miR-1983*), 002306 (*mmu-miR-214*) and 001973 (*U6* snRNA).

### Gene Set Enrichment Analysis of mRNA array data

For Gene Set Enrichment Analysis, we used the Bioconductor package limma’s *romer* function, which boosts the signal-to-noise ratio and performs a cross-correlation analysis to detect sets of genes in which the constituents show coordinated changes in expression [36–38]. To be stringent, only gene sets meeting the criterion of P < 0.01 were considered, and qualifying gene sets were cross-tabulated to exclude sets that were duplicated or that were subsets of a larger qualifying gene set. We used the Broad Institute’s Molecular Signatures Database (MSigDB) collection of >10,000 gene sets divided into 7 major collections, and we chose two major collections (C2 and C5) which were curated genes (subsets Biocarta, Reactome), and the Gene Ontology (GO) gene sets (subsets: cellular component, molecular function and biological process) respectively (http://www.broadinstitute.org/gsea/msigdb/index.jsp).

### Identification of miR targets

The miR target feature of the Ingenuity Pathway Analysis software was used to identify miR targets (http://www.ingenuity.com/, Version 18030641 (Release Date: 2013-12-06)). IPA uses the TargetScan database for predicted miR-mRNA targets. TargetScan uses seed paring to predict mRNA targets. High predicted confidence is assigned either if the relationship is between a conserved or highly conserved miR as defined by TargetScan and at least one conserved site on the targeted sequence or the total context score, as defined by TargetScan, is -0.4 or less. This indicates that the miR is predicted to repress the expression of its mRNA target to 40% of the "normal" level. Moderate predicted confidence is assigned if the total context score, as defined by TargetScan, is -0.2 or less. This indicates that the miR is predicted to repress the expression of its mRNA target to 65% of the "normal" level. The miR targets presented below were all predicted with high or moderate confidence.

### Public database

All gene expression data have been deposited to the National Center for Biotechnology Information Gene Expression Omnibus public database repository (http://www.ncbi.nlm.nih.gov/geo/). The data is accessible under GEO accession number GSE62346.

## Results

**Fig 1:**
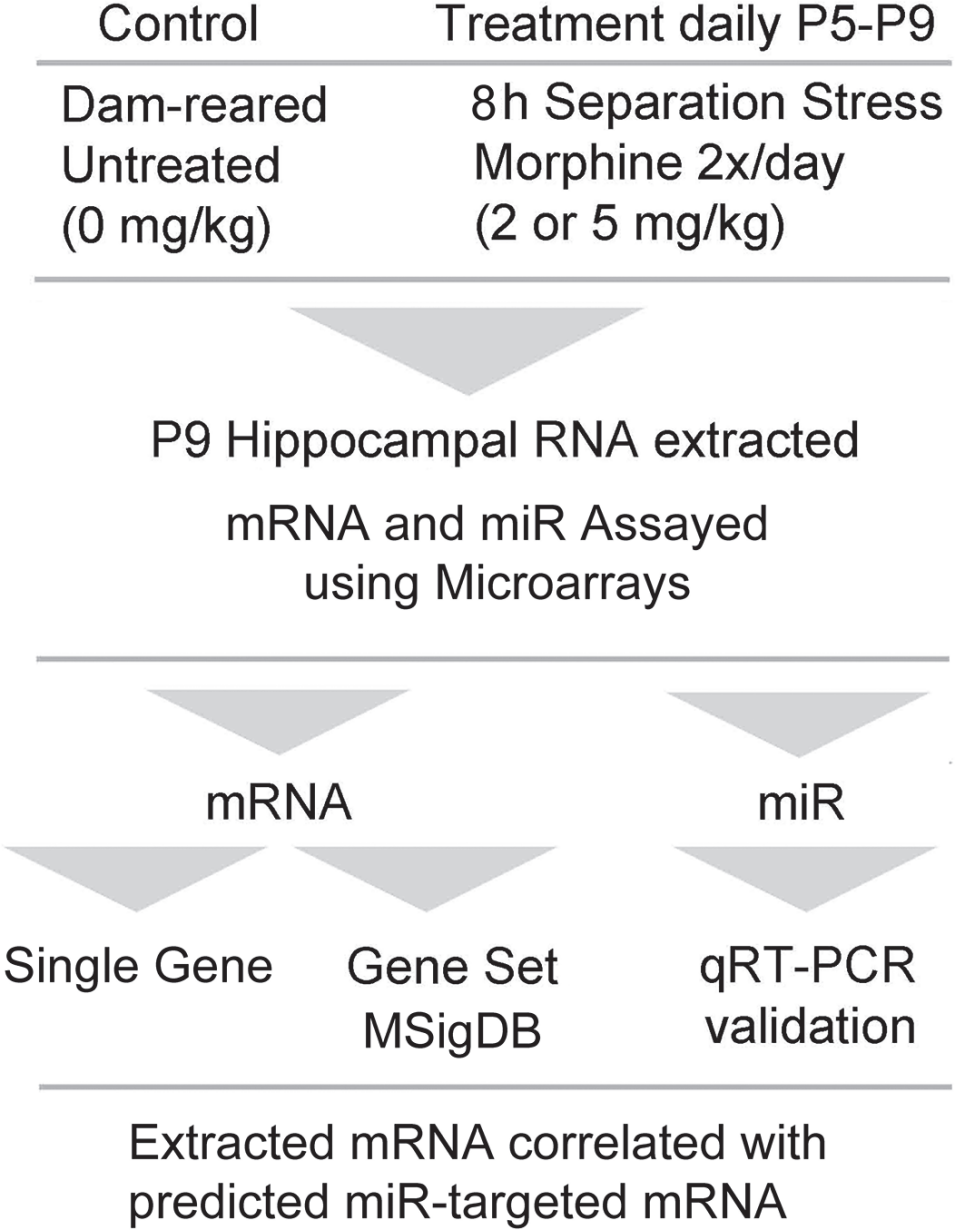
Flow diagram illustrating the experimental design and analysis. Neonatal mice were treated from postnatal day five (P5) to P9 as described and RNA was analyzed as shown. Abbreviation: molecular signatures database (MSigDB).

To assist the reader with interpretation of the data, Fig 1 is a flow diagram illustrating the general experimental design and analysis.

### Single Gene Analysis

**Fig 2:**
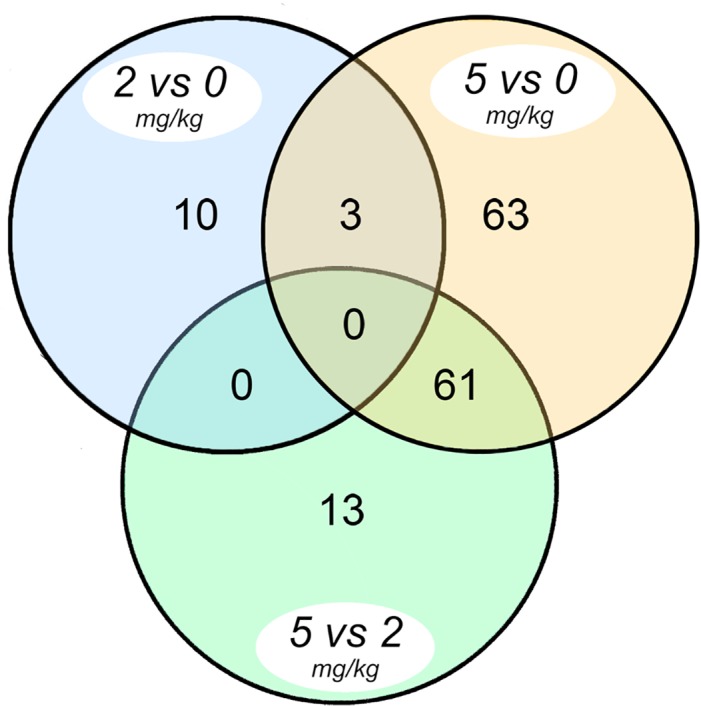
Venn diagram showing differential expression (>1.5-fold, p ≤ 0.05) of mRNAs that are unique as well as those shared by the three morphine dose contrasts indicated.

Morphine produced differential expression (>1.5 fold, p<0.05) of neonatal mouse hippocampal genes. Overall, 150 of 21,045 probesets exhibited differential expression and Fig 2 is a Venn diagram illustrating the comparative distribution of those effects. From these 150 probesets, 100 unique genes with functionally meaningful annotations were recognized (21 genes were up- and 79 genes were down-regulated). Select morphine-responsive mRNAs (p<0.05) are listed in Table 1 and are sorted based on fold change. Seven of the genes most affected by morphine, as determined by microarray, were also quantified by qPCR (highlighted in Table 1) and those data were analyzed using dose of morphine as a continuous covariate. Five of the seven genes re-quantified using qPCR exhibited corroborating changes in expression that also varied significantly as a function of morphine dose. The entire single gene analysis and a heat map are available in (S1 Table and S1 Fig).

**Table 1:** Single Genes affected by MS5.

Symbol	Systematic Name (NM)	mRNA fold change log_2_ compared to Control		Protein
		MS2	MS5	
*Defb11* ^*a*^	139221	-0.16	-2.11**	defensin beta 11
*Aqp1* ^*b*^	007472	0.16	-1.79*	aquaporin 1
*Calml4* ^*c*^	138304	-0.27	-1.71**	calmodulin-like 4
*Cldn2* ^*d*^	016675	-0.10	-1.52**	claudin 2
*Folr1*	008034	-0.23	-1.48**	folate receptor 1
*Slc4a5*	1166067	0.06	-1.47**	solute carrier 4, 5
*Sulf1*	172294	-0.18	-1.34**	sulfatase 1
*Kl*	013823	-0.27	-1.28**	klotho
*Car14*	011797	-0.12	-1.10**	carbonic anhydrase 14
*Msx1*	010835	-0.14	-1.01**	msh homeobox 1
*Sln*	025540	0.18	-0.90*	sarcolipin
*F5*	007976	-0.10	-0.77 **	coagulation factor V
*Defb35*	139224	-0.28 *	-0.76 **	defensin beta 35
*Tgfbi*	009369	-0.09	-0.73 **	transforming growth factor, beta
*Pcolce2*	029620	-0.12	-0.72 **	procollagen C proteinase enhancer 2
*Hoxb3*	1079869	-0.17	-0.64 **	homeobox B3
*Loxl1*	010729	-0.11	-0.63 **	lysyl oxidase-like 1
*Defb10*	139225	-0.01	-0.60 **	defensin beta 10
*Itgax*	021334	-0.44 *	-0.60 **	integrin alpha X
*Tcea3*	011542	0.07	-0.60 **	transcription elongation factor A (SII)
*Cdh7*	172853	0.16	0.61*	cadherin 7, type 2
*Epha10*	177671	0.33	0.63*	Eph receptor A10
*Zmat4*	177086	0.36	0.67*	zinc finger, matrin t4
*Oprk1*	011011	-0.04	0.81*	opioid R, kappa1
*Fign* ^*e*^	021716	-0.14	1.52**	Fidgetin
*Trh*	009426	-0.20	1.98**	thyrotropin-releasing hormone
*Avp* ^*f*^	009732	0.30	2.45*	arginine vasopressin
*Retnla* ^*g*^	020509	0.37	2.58**	resistin like alpha

Selected single genes exhibiting differential microarray gene expression in hippocampus taken from neonatal mice exposed to a mild stress and morphine (2 or 5 mg/kg) protocol. Superscripts (a-g) highlight seven of the genes most affected whose expression patterns were also quantified by qPCR and analyzed using morphine dose as a covariate. Genes are ordered by increasing fold change of MS5.

Array P-values: * P ≤ 0.05

** P ≤ 0.01; qPCR P-values

^*a*^ 0.01

^*b*^ 0.02

^*c*^ 0.01

^*d*^ 0.05

^*e*^ 0.58

^*f*^ 0.04

^*g*^ 0.45.

### Gene Set Enrichment Analysis (GSEA)

Gene sets and pathways with concordant changes in expression were identified using separate Romer analyses based on the GO:C5 (cell/molecular/biological), Biocarta and Reactome gene set collections. Tables 2–6 list the gene sets that are significantly altered at p<0.01 in mouse hippocampus following neonatal morphine exposure (MS5 *vs*. controls) and complete listings are provided in (S2–S6 Table). Upregulated gene sets in the MS5 group were frequently related to cell signaling/synaptic transmission, cyclic AMP signaling, glutamate receptor activity, and regulation of lymphocyte activation. Gene sets downregulated following MS5 exposure were frequently related to transmembrane transport and cellular development. Biocarta GSEA identified effects of morphine on immunomodulation pathways including beta-arrestin and T cell signaling, death domain, nuclear factor kappa B (NF-kB), and stress-related signaling. Reactome GSEA found notable effects of morphine on neurotransmission including upregulation of GABA and opioid signaling, and downregulation of NOTCH, apoptosis, and lipid and carbohydrate metabolism.

**Table 2:** GO:C5 MS5-upregulated mRNA pathways.

Gene Set Description	P	# Up	# Down
*Cellular Component*	(none met criterion P<0.01)		
*Molecular Function*			
3',5'-cAMP Phosphodiesterase Activity	0.003	11	2
Cyclic Nucleotide Phosphodiesterase Activity	0.004	12	2
N-methyltransferase Activity	0.001	8	5
Oxidoreductase Activity	0.008	18	12
Phosphatase Inhibitor Activity	0.001	10	1
Phosphatase Regulator Activity	0.009	19	6
Serotonin Receptor Activity	0.001	7	3
SH2 Domain Binding	0.005	11	4
Sodium Channel Activity	0.008	10	5
Transcription Elongation Regulator Activity	0.009	7	3
*Biological Process*			
B Cell Activation	0.008	11	8
Behavior	0.003	76	57
Calcium-mediated Signaling	0.003	11	4
cAMP-Mediated Signaling	0.004	40	23
Cell Activation	0.007	38	30
Central Nervous System Development	0.0002	64	53
Chromosome Organization and Biogenesis	0.003	7	3
Endothelial Cell Proliferation	0.0001	7	3
G Protein Receptor Pathway	0.0004	175	142
Hormone Secretion	0.002	12	4
Leukocyte Differentiation	0.010	21	14
Lymphocyte Activation	0.010	13	7
Multicellular Organismal Process	0.0002	76	62
Muscle Contraction	0.0001	13	5
Myeloid Cell Differentiation	0.005	11	6
Negative Regulation of Catalytic Activity	0.003	37	28
Neurological System Process	0.004	201	152
Positive Regulation of Cell Adhesion	0.001	8	3
Regulation of Homeostasis	0.003	8	4
Response to Extracellular Stimulus	0.007	18	14
Second Messenger-mediated Signaling	0.001	85	61

Gene sets (GO:C5) with mRNAs exhibiting upregulated expression due to neonatal morphine (MS5) exposure. Alphabetical order.

**Table 3:** GO:C5 MS5-downregulated mRNA pathways.

Gene Set Description	P	# Up	# Down
*Cellular Component*			
Early Endosome	0.003	4	13
Integrin Complex	0.004	4	15
Ruffle	0.005	8	23
*Molecular Function*			
Amino Acid Transmembrane Transport	0.004	8	20
Beta Tubulin Binding	0.002	1	8
Carbohydrate Kinase Activity	0.009	3	12
CH-CH Oxidoreductase Activity	0.001	3	18
Collagen Binding	0.008	3	10
DNA Polymerase Activity	0.006	4	14
Identical Protein Binding	0.0001	109	182
Metalloexopeptidase Activity	0.008	2	11
Organic Acid Transmembrane Transport	0.003	12	31
Peptidase Activity	0.0001	60	101
Protease Inhibitor Activity	0.008	10	20
Protein Dimerization Activity	0.002	69	101
Transition Metal Ion Binding	0.008	34	63
Translation Initiation Factor Activity	0.0001	1	22
Unfolded Protein Binding	0.007	11	29
*Biological Process*			
Angiogenesis	0.005	0	13
Anion Transport	0.009	7	20
Bile Acid Metabolic Process	0.003	2	8
Carbohydrate Response	0.004	3	8
Cell Adhesion	0.008	33	50
Cell Division	0.005	4	16
NF-κB Cascade	0.007	40	67
Nucleotide Sugar Metabolism	0.004	2	8
Organ Morphogenesis	0.007	50	90
Peptide Metabolic Process	0.005	2	7
Proteolysis	0.001	67	111
Tube Development	0.001	5	13
Vitamin Transport	0.009	1	10

Gene sets (GO:C5) with mRNAs exhibiting downregulated expression due to neonatal morphine (MS5) exposure. Alphabetical order.

**Table 4:** GO:C5 MS5-mixed expression mRNA pathways.

Gene Set Description	P	# Up	# Down
*Cellular Component*			
Cell Cortex Part	0.008	4	12
Extracellular Region	0.008	170	225
*Molecular Function*			
Histone Acetyltransferase Activity	0.005	6	5
Hydrolysis of Glycosyl Bonds	0.002	15	26
Hydrolysis of Glycosyl Compounds	0.002	13	18
*Biological Process*			
Aging	0.001	5	6
Cell Proliferation	0.008	52	95
DNA Replication	0.003	5	14
Glutamine Family Metabolism	0.006	9	4
Heme Biosynthetic Process	0.003	4	6
Homeostatic Process	0.008	85	105
Signal Transduction	0.001	91	121

Gene sets (GO:C5) with mRNAs exhibiting mixed up/down expression due to neonatal morphine (MS5) exposure. Alphabetical order.

**Table 5:** Biocarta MS5-responsive Gene sets.

Pathway (Biocarta)	P	# Up	# Down
*UP*			
β-Arrestin Src	0.0001	13	1
CDK5	0.0022	8	2
CSK	0.0027	14	5
ERK	0.0001	17	10
GLEEVEC	0.0065	15	7
HER2	0.0018	16	4
IGF1R	0.0029	13	8
MAL	0.0015	12	5
NO2-dep IL12	0.0044	14	3
*DOWN*			
ACE2	0.0023	2	9
AHSP	0.0015	2	8
ARF	0.0015	4	12
CARM1	0.0027	7	5
MTA3	0.0005	4	13
PTC1	0.0002	4	7
TGFB	0.0046	11	8
VITCB	0.0081	3	8
*MIXED*			
AMI	0.0023	3	17
DEATH	0.0015	9	21
NF-κB	0.0057	4	19
RB	0.0003	1	11
SODD	0.0002	1	9

Gene sets (Biocarta) with mRNAs affected by neonatal morphine (MS5). Alphabetical order.

**Table 6:** Reactome MS5-responsive Gene sets.

Pathway (Reactome)	P	# Up	# Down
*UP*			
Aquaporin Mediated Transport	0.004	31	17
Botulinum Neurotoxicity	0.002	12	3
Circadian Clock	0.003	30	20
FGFR Signaling	0.001	26	15
GABA Receptor Activation	0.006	37	15
Gastrin-CREB Signaling	0.008	108	83
Glucagon Regulation of Insulin	0.002	29	12
G-Protein Signaling	0.004	249	195
Interaction: L1 and Ankyrins	0.003	11	2
Interleukin 2 Signaling	0.001	23	16
Interleukin 3 Signaling	0.005	24	17
Netrin1 Signaling	0.007	6	3
Neuronal System	0.004	177	91
Nitric Oxide Signaling	0.004	15	10
Opioid Signalling	0.003	49	26
Phosphorylation of CD3 and TCR Zeta	0.007	8	1
Potassium Channels	0.004	60	37
*DOWN*			
AKT1-mediated Events	0.001	7	21
Apoptosis Extrinsic Pathway	0.0002	0	11
Calcium-mediated Platelet Response	0.007	26	49
Caspase-mediated Protein Cleavage	0.004	3	8
Chaperonin-mediated Biosynthesis	0.0001	4	22
Fatty Acid Metabolism	0.004	60	103
Integrin Cell Surface Interactions	0.009	23	54
Lipid Digestion and Transport	0.0003	9	33
Metabolism of Carbohydrates	0.007	78	144
Metabolism of Porphyrins	0.003	3	10
Notch Receptor Cleavage	0.002	4	7
Transmembrane Transport	0.01	87	146
*MIXED*			
DCC-mediated Attractive Signaling	0.003	7	6
Extracellular Matrix Organization	0.006	24	58
Meiotic Recombination	0.009	13	40
Methionine and cysteine Metabolism	0.007	7	17
PDGF Signaling	0.005	51	63
Pre-Notch Expression	0.003	15	25

Gene sets (Reactome) with mRNAs affected by neonatal morphine (MS5). Alphabetical order.

**Fig 3:**
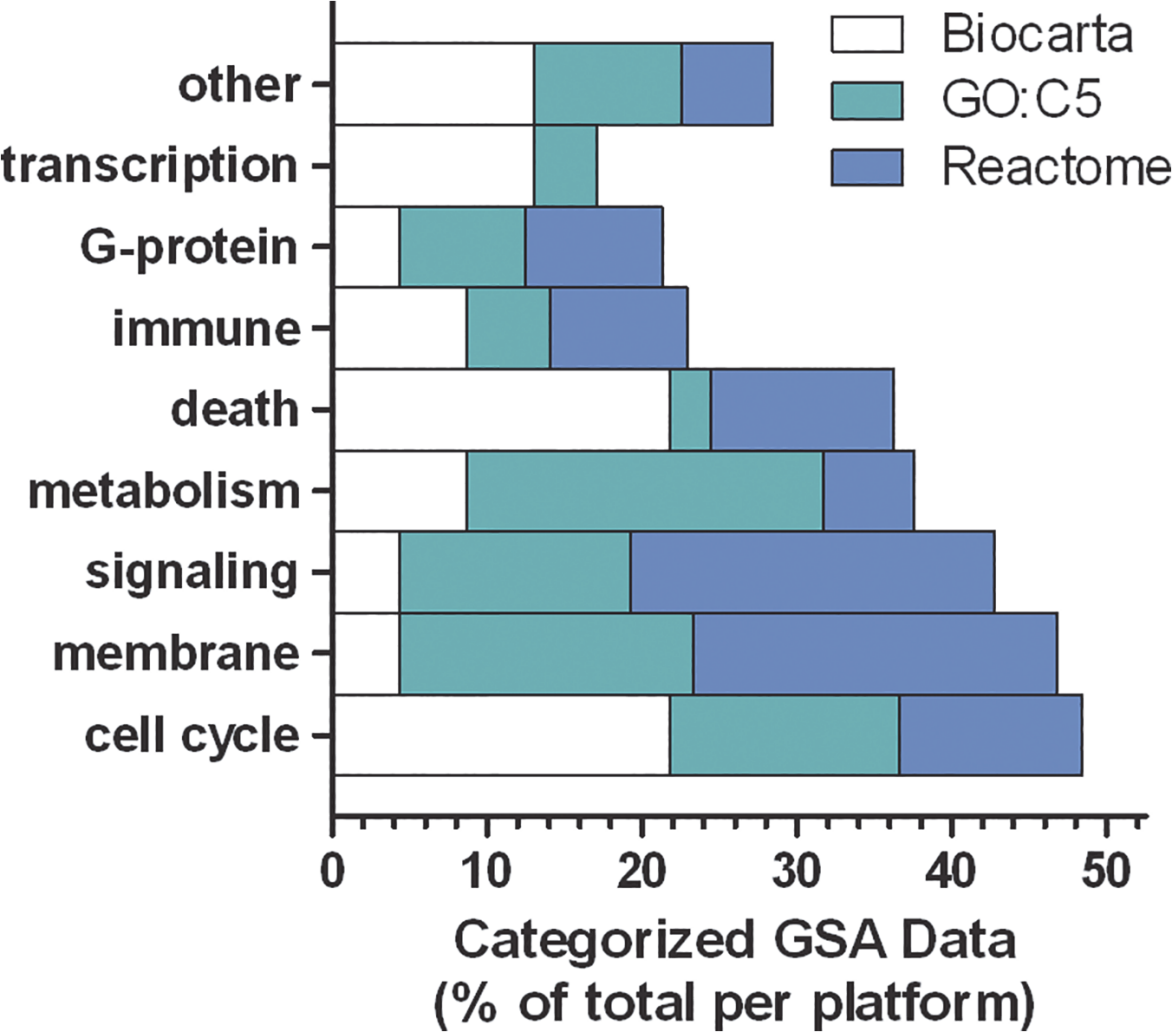
Categorized Gene Set Enrichment Analysis (GSEA) data to contrast the effects of morphine detected using three separate gene set collections. Individual GSEA data points from Tables 2–6 were assigned to basic biological function categories and the counts for each category were divided by the total for each gene set collection (Reactome, dark gray; GO:C5, light gray; Biocarta, white). This illustrates the relative agreement between gene set collections used to detect differential gene expression after neonatal morphine treatment.

To combine the GSEA data from the three gene set collections, an ensemble analysis was performed by defining 9 categories of biological functions and assigning each gene set pathway identified in Tables 2–6 to a category, then the categorized data points were counted and divided by the total to create proportions. The resulting data in Fig 3 illustrate that morphine’s effects on hippocampal gene expression predominantly affected processes related to cell cycle, membrane biology, signaling, metabolism and cell death.

### miR microarrays

**Fig 4:**
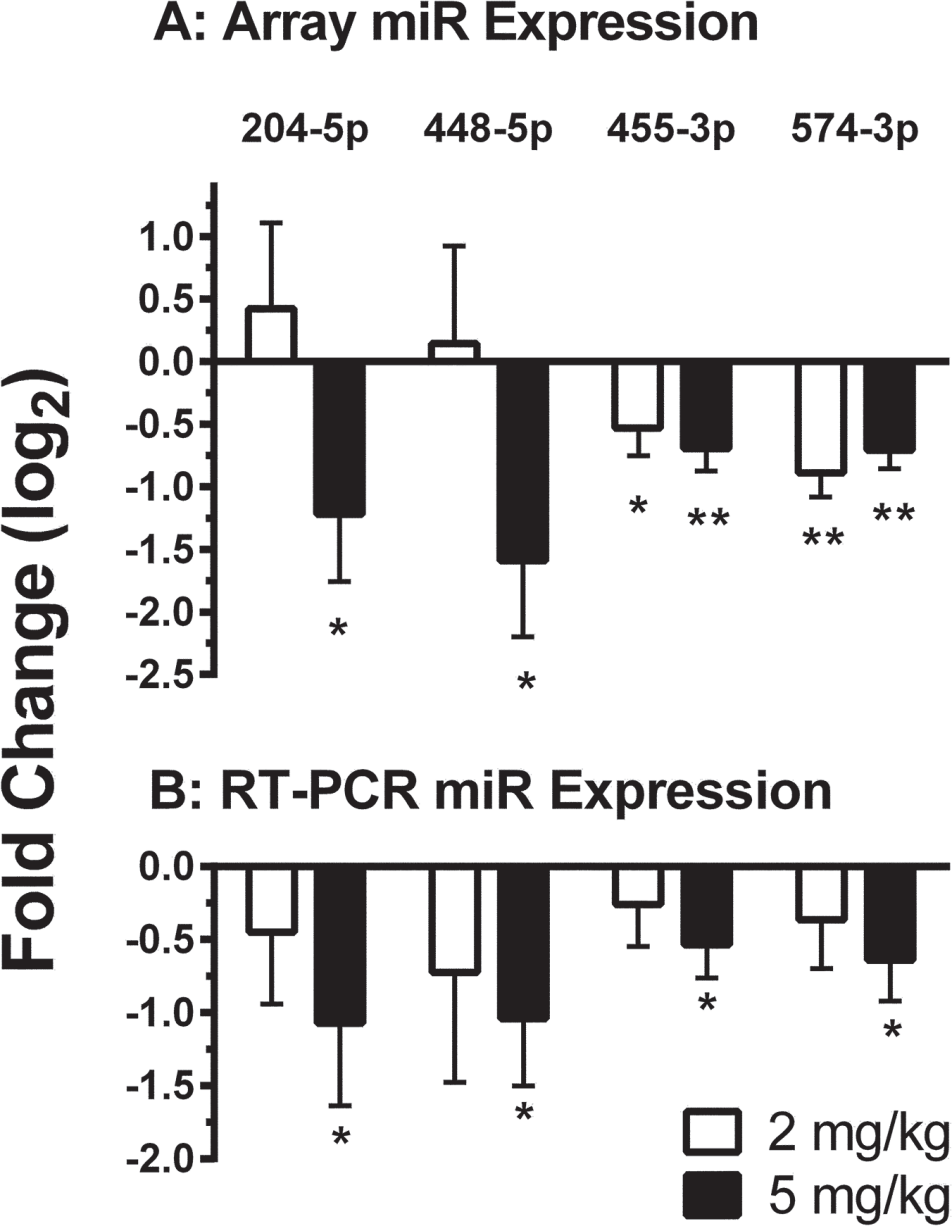
Hippocampal miR expression in stressed neonatal mice was down-regulated by morphine treatment. Data are mean (+SEM) microarray expression (panel A) and RT-PCR expression (panel B) values presented as fold-change (log_2_) compared to untreated control mice. Values from mice receiving either 2 mg/kg morphine (unfilled bars) or 5 mg/kg morphine (filled bars) are shown. Expression values that differ significantly from control (0) are indicated as * or ** = *P* ≤ 0.05 or 0.01, respectively.

To investigate whether effects of neonatal morphine on hippocampal mRNA expression in stressed mice are secondary to effects on miR expression, morphine-responsive miRs were identified. miR array profiling identified ten specific miRs that were altered (>1.5 fold, p < 0.05) by morphine (*miR-204*, *miR-448*, *miR-455*, *miR-574*, *miR-34c*, miR-34c*, *miR-1839*, *miR-153*, *miR-1983* and *miR-214*) and RT-PCR analysis was performed for those ten miRs. The first four in that list were validated by quantitative RT-PCR. Fig 4 illustrates the dose-dependent effects of morphine on array expression (panel A) and RT-PCR expression (panel B) of *miR-204-5p*, *miR-448-5p*, *miR-455-3p*, and *miR-574-3p*. Three of those four (*not miR-574-3p*) validated miRs had mRNA targets that were also differentially expressed and Table 7 presents the morphine-responsive miRs along with their corresponding mRNA targets. These data suggest that neonatal morphine may affect both mRNA and associated miR expression in mouse hippocampus.

**Table 7:** Hippocampal miR and miR-targeted mRNA expression was altered in neonatal mice exposed to stress plus morphine (MS5) compared to control.

miR	Fold change log2	*P*_miR_	Targeted mRNA	Fold change log2	*P*_mRNA_	mRNA product
*204-5p*	-1.222	0.049	*Ap1s2*	-0.764	0.002	adaptor complex 1 σ2 ^a^
			*Syt6*	0.97	0.047	synaptotagmin VI ^a^
*448-5p*			*Acox2*	-0.927	0.011	acyl-CoA oxidase 2 ^b^
			*Kl*	-1.108	0.006	klotho ^b^
	-1.589	0.024	*Otx2*	-1.257	0.031	orthodenticle homeobox 2 ^c^
			*Prdm16*	-0.601	0.023	PR domain containing 16 ^c^
			*Sema3A*	0.599	0.043	semaphorin domain 3A ^d^
*455-5p*			*Ap1s2*	-0.764	0.002	adaptor complex 1 σ2 ^a^
	-0.695	0.004	*Pdzd2*	0.596	0.049	PDZ domain 2 ^e^
			*Prg4*	0.798	0.014	proteoglycan 4 ^f^

All targets are either high or moderate predicted in IPA. protein categories

^a^ transporter protein

^b^ enzyme

^c^ transcription regulator

^d^ immunoglobulin

^e^ neurotransmission

^f^ proteoglyclan. Alphabetical order.

## Discussion

Neonatal morphine exposure produced a variety of changes in mouse hippocampal gene expression. Moreover, neonatal morphine produced dose-dependent suppression of four specific miR sequences. Three of these four miRs targeted nine morphine-responsive mRNA genes. These data support the hypothesis that some morphine-induced changes in brain gene expression involve modulation of miR-mediated mRNA regulation. Although this study is a descriptive microarray analysis, these data provide a foundation for future specific hypothesis testing about possible side effects of morphine exposure during development.

The current data also raise concerns about possible deleterious effects of neonatal morphine exposure because more than 100 single genes and gene set pathways were affected. The ensemble analysis in Fig 3 identified that neonatal morphine exposure predominantly affected cell cycle-, membrane-, signaling-, and metabolism-related functions. In a developing brain, those functions are essential for proper proliferation, differentiation and migration and, therefore, morphine’s modulation or disruption of those processes could be very detrimental. This concern is validated by the fact that morphine inhibits adult neurogenesis and alters neuronal phenotypes in the rat hippocampus [39, 40].

Clinically, morphine may be prescribed to sedate infants so as to alleviate pain and stress during intensive care. Stress and morphine treatment affect hippocampal gene expression in rodents [11]. To understand effects of neonatal stress on brain development, we previously examined differential hippocampal mouse gene expression to compare multiple levels of neonatal stress (mild *vs*. severe) combined with 2 mg/kg morphine cotreatment [11]. As a follow-up, the current experiment compared multiple doses of neonatal morphine (2 *vs*. 5 mg/kg) given to severely stressed mice. In both experiments, differential single gene expression in morphine-treated mice was detected for *aquaporin*, *claudin*, *cadherin*, *and arginine vasopressin*, but in the current mice given 5 mg/kg morphine, the magnitude of the effects were either enhanced or profoundly reversed. This interaction agrees with findings from the prior experiment in which morphine treatment did *not* simply attenuate effects of neonatal stress on gene expression but, instead, additional effects were evident when morphine and stress were combined. Collectively, it is now apparent that neonatal morphine exposure produces dose-dependent changes in hippocampal gene expression when administered to treat stress.

Morphine can suppress the immune response in animals and humans [41–46]. Consistent with this immune inhibition, we found that morphine greatly suppressed β-defensin 11 (*Defb11)* mRNA levels, a factor known to mediate innate/adaptive immune and inflammatory responses. Given that hyperglycemia occurs during neonatal stress [47], and that hyperglycemia-induced neurodegeneration is also associated with *β*-defensin suppression [48], we speculate that neonatal morphine-induced *β*-defensin suppression could increase a neonatal animal’s susceptibility to neurodegenerative injury, particularly under conditions of stress. The immunoregulatory effects of morphine may involve gene pathways, such as the morphine-mediated enhancement of ERK phosphorylation and inhibition of NF-κB signaling seen in activated human T cells [49]. Consistent with this morphine-mediated pathway regulation, in our MS5 mice, the ERK pathway was upregulated (see Table 5) and the NF-κB gene pathway was downregulated (see Tables 3 and 5) in the hippocampus.

Stimulatory immune-related effects of chronic morphine include increasing circulating T-regulatory and T-helper (Th) 17 cells [50], promoting GATA 3 and T-bet transcription factor expression to increase IL-4, IL-5 and Th2 effector cells [42] and increasing IL-12 production in mouse and human cells [43, 51]. Congruent with these effects, we found that neonatal morphine stimulated Th, T-cytotoxic, beta-arrestin, IL-5, and IL-12-related pathways (Table 5, Biocarta), and also increased the lymphocyte activation pathway (Table 6, Reactome). Recognizing that the neonatal immune response is normally limited to Th2 activity,[52] the artificial elevation of these factors in neonatal brain could be deleterious if it promoted brain inflammation. In fact, an elevated immune response in neonatal brain is suspect in early white matter injury [53].

The most novel feature of this study is that it included an analysis of miRs. We hypothesize that effects of morphine on miR expression could have lasting neuroepigenetic consequences. During early human brain development, changes in the temporal or spatial patterns of miR expression could result in epigenetic modifications that effectively alter differentiation. Throughout early human brain development, there is activity in miR-sensitive gene sets related to autism, schizophrenia, bipolar disorder and depression [54]. Thus there is a strong rationale for testing the hypothesis that neonatal morphine exposure may trigger permanent miR-mediated epigenetic changes, and this hypothesis provides a mechanism for understanding possible neonatal origins of childhood or adult neurologic diseases.

In our neonatal mice, three miRs, *miR-448-5p*, *mir-204-5p*, and *miR-455-5p*, which targeted 9 of the 63 experimentally determined differentially expressed mRNAs, exhibited decreased expression in MS5-treated hippocampus. The first miR, *miR-448-5p*, was matched to 5 of the morphine-responsive mRNAs (*Sema3a*, *Prdm16*, *Acox2*, *Kl*, *Otx2*) whose products are related to neurodevelopment and inflammation. For example, semaphorin 3A (*Sema3a*), was up-regulated and is associated with axonal branching [55]. Orthodenticle homeobox 2 (*Otx2*), Klotho (*Kl*), acyl-CoA oxidase 2 branched chain (*Acox2*), and PR domain-containing 16 (*Prdm16*) were all down-regulated. The homeobox Otx2 plays a significant role in brain development and function of mesencephalic dopaminergic neurons [56–58]. Moreover, Klotho is a novel β-glucoronidase transmembrane enzyme that is highly expressed in both choroid plexus and hippocampus where it produces anti-inflammatory [59, 60] and antioxidative effects, and also controls calcium and phosphate homeostasis [61]. In fact, Klotho-deficient mice demonstrate hippocampal neuronal degeneration and decreased cortical Purkinje cell body density [62]. The *Acox2* gene encodes for the branched-chain acyl-CoA oxidase protein, and deficiency of this enzyme results in the accumulation of branched fatty acids and may lead to Zellweger syndrome, severe mental retardation, and death in children [63]. In mice, the transcription factor Prdm16 is required for neural stem cell survival, cell cycle regulation, and self-renewal, which occurs partially by promoting Hepatocyte Growth Factor expression and regulating ROS levels [64].

The sequence *mir-204-5p* targeted 2 mRNA genes (*Syt6* and *Ap1s2*) and expression of the synaptotagmin (*Syt6*) was increased while adaptor-related protein complex 1, sigma 2 subunit (*Ap1s2*) was decreased. Synaptotagmins are abundant, evolutionarily conserved integral membrane proteins that mediate calcium-dependent exocytosis and neurotransmitter release [65, 66]. *Ap1s2* encodes the σ-2 subunit of the early transcription factor *Ap1* (*c-Jun*) [67] and the product Jun N-terminal kinase is associated with synaptic protein trafficking [68, 69]. Mutation of *AP1S2* protein is associated with X-linked Dandy–Walker malformation with intellectual disability, basal ganglia disease and seizures (Pettigrew syndrome) [67].

The miR *mir-455-5p* was associated with 3 altered mRNAs (*Prg4*, *Pdzd2*, *and Ap1s2*). The *miR-455-5p* was decreased and both *Prg4* and *Pdzd2* were correspondingly increased as expected based on classical miR-mRNA regulation. However, despite the suppression of both *mir-204-5p* and *miR-455-5p*, *Ap1s2* was still decreased. It is difficult to know whether the patterns of expression reflect a response to morphine, or a response to changes in expression of the other morphine-responsive factors. Given that Ap1/Jnk is associated with hippocampal apoptosis [70, 71], the decrease may be a welcome outcome. The *Prg4* gene encodes a proteoglycan that is increased in mice following experimental middle cerebral artery occlusion [72]. The *Pdz2* gene product regulates expression of Nav1.8 cytokine-modulated tetrodotoxin-resistant sodium channels [73, 74].

There are limitations to descriptive microarray studies that should be mentioned. First, the current databases relating mRNA genes and miR sequences may be refined in the future. Regarding interspecies conservation, the relationships between mRNA/miR in humans may be different than in mice [75]. Microarray effects on expression are descriptive and do not indicate whether post-transcriptional regulatory mechanisms are involved. We presume that the principal effects of morphine identified were due to direct action of morphine on hippocampal opioid receptors, but we cannot exclude the possible indirect involvement of non-hippocampal loci. We identified miR and mRNA that were altered in hippocampal tissue, but we did not identify the specific cell types expressing these molecules. Additionally, varying levels of stress, the timing of stress and morphine exposure, the concentration, dosage, and duration (acute vs chronic) of morphine, and sex-specific differences may influence hippocampal miR:mRNA interactions. We also cannot comment on the influence of stress and morphine on miR expression in other regions of the brain that may affect neurodevelopment.

## Conclusion

Repeated neonatal morphine exposure altered gene expression in the hippocampus of stressed mice, affecting pathways involved in cell cycle, membrane function, signaling, metabolism, cell death, and immune regulation. Morphine also induced a dose-dependent down-regulation of *miR-448-5p*, *mir-204-5p*, and *miR-455-5p* expression with corresponding changes in their mRNA targets involved in neurodevelopment, neurotransmission, and inflammation. If morphine–mediated neuroepigenetic effects are permanent and affect long-term hippocampal function, this would suggest one potential mechanism occurring in the neonatal period that may lead to future neurodevelopmental impairment. A better understanding of the effects of morphine on mRNA and miR expression may lead to novel pharmacological approaches to provide sedation without deleteriously altering neurodevelopmental outcomes.

## Supporting Information

S1 Fig A heat map illustrating single gene differential mRNA expression in neonatal mouse hippocampal tissue. (TIF)

S1 Table Single gene array expression dataset. (XLSX)

S2 Table Romer Cellular Component gene set analysis comparing 5 mg/kg morphine (trt5) to control (trt0). (XLSX)

S3 Table Romer Biological Process gene set analysis comparing 5 mg/kg morphine (trt5) to control (trt0). (XLSX)

S4 Table Romer Molecular Function gene set analysis comparing 5 mg/kg morphine (trt5) to control (trt0). (XLSX)

S5 Table Biocarta gene set analysis comparing 5 mg/kg morphine (trt5) to control (trt0). (XLSX)

S6 Table Reactome gene set analysis comparing 5 mg/kg morphine (trt5) to control (trt0). (XLSX)
