# Supplementary figures and images for: Dose-Dependent Effects of Morphine Exposure on mRNA and microRNA (miR) Expression in Hippocampus of Stressed Neonatal Mice
Source: PLoS One. 2015 Apr 6;10(4):e0123047. doi: 10.1371/journal.pone.0123047 (PMC4386824; doi:10.1371/journal.pone.0123047)

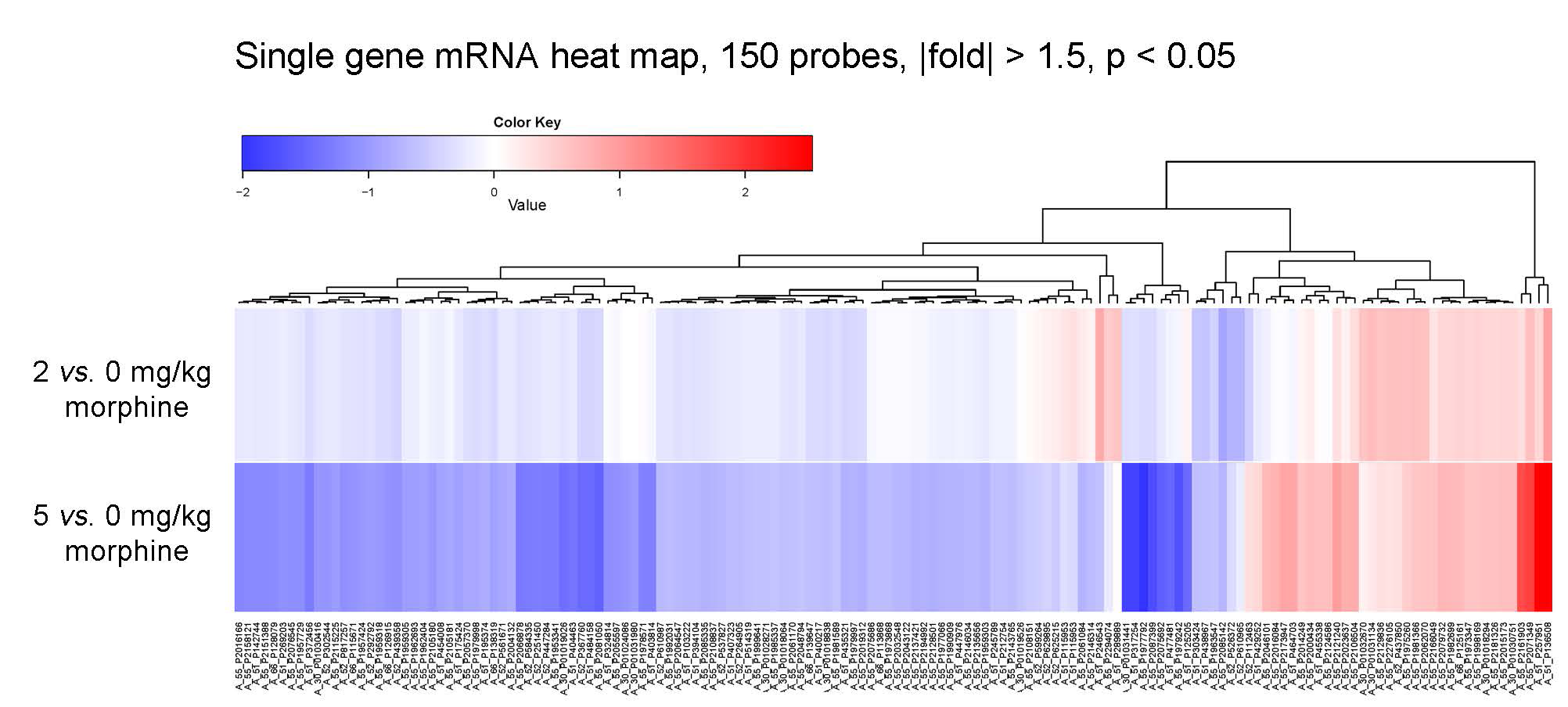

Supplement: S1 Fig — (TIF) [file pone.0123047.s001.tif]
